# Effectiveness of nutritional supplementation during the first 1000-days of life to reduce child undernutrition: A cluster randomized controlled trial in Pakistan

**DOI:** 10.1016/j.lansea.2022.100035

**Published:** 2022-07-19

**Authors:** Sajid Bashir Soofi, Gul Nawaz Khan, Shabina Ariff, Yasir Ihtesham, Mahamadou Tanimoune, Arjumand Rizvi, Muhammad Sajid, Cecilia Garzon, Saskia de Pee, Zulfiqar A. Bhutta

**Affiliations:** a Department of Paediatrics and Child Health, Aga Khan University, Karachi, Pakistan; b Centre of Excellence in Women and Child Health, Aga Khan University, Karachi, Pakistan; c World Food Programme, Islamabad, Pakistan; d World Food Programme, Italy

**Keywords:** Wheat soya blend plus, Lipid-based nutrient supplement, Stunting, Underweight, Wasting

## Abstract

**Background:**

Childhood stunting can start in the womb and continue for two years. Therefore, the first 1000 days of life between a woman's pregnancy and her child's 2nd birthday offer a unique window of opportunity to build healthier and more prosperous futures. Therefore, we aimed to assess the effectiveness of nutritional supplementation during the first 1000-days to reduce the prevalence of stunting in children at 24 months of age.

**Methods:**

In this cluster randomized controlled trial, we enrolled women during their pregnancy from two rural districts of Sindh, Pakistan. A cluster was one union council with a population of ∼25000 residents. Out of 29 clusters, we randomly allocated 6 clusters to the intervention and control groups each. Pregnant women received a monthly supply of 5 kg (i.e., 165 grams/day) of wheat soya blend plus (WSB+) during pregnancy and the first six months of their lactation period. In addition, their children received lipid-based nutrient supplement - medium-quantity (LNS-MQ) between 6-23 months of age. The primary outcome was a reduction in the prevalence of stunting in children at 24 months of age. Analysis was an intention to treat. The trial is registered on ClinicalTrial.gov, number NCT02422953.

**Findings:**

Two thousand thirty pregnant women (1017 in the intervention group and 1013 in the control group) were enrolled between August 30, 2014, and May 25, 2016. Monthly follow-ups were conducted between October 1, 2014, and October 25, 2018. At 24 months of age, we captured data from 699 (78%) of 892 live births in the intervention group and 653 (76%) of 853 live births in the control group. There was a significant difference in mean length (49.4 cm vs 48.9 cm, *p =*0.027), weight (3.1 kg vs 3.0 kg, *p =*0.013), length for age z-scores (-1.2 vs -1.5, *p =*0.004) and weight for age z-scores (-1.2 vs -1.5, *p =*0.015) among infants in the intervention compared to control group. At 24 months of age, a significant difference in the prevalence of stunting (absolute difference, 10.2%, 95% CI 18.2 to 2.3, *p =*0.017) and underweight (absolute difference, 13.7%, 95% CI 20.3 to 7.0, *p =*0.001) were observed in the intervention as compared to the control group. The prevalence of wasting was not significantly different between the intervention and control groups (absolute difference, 6.9%, 95% CI 14.1 to 0.3, *p =*0.057).

**Interpretation:**

Provision of WSB+ and LNS-MQ during the first 1000-days of life improved child linear growth and reduced stunting in children at 24 months. This study can be scaled-up in similar settings to lower the prevalence of stunting in children under two years of age.

**Funding:**

World Food Programme, Pakistan

**Research in context**

**Evidence before this study**

When this study was conceived in 2014, evidence linking the effectiveness of nutritional supplementation during the first 1000 days of life on reducing the prevalence of stunting in children at 24 months was scarce. At the time of study design, we used the 2013 Lancet Series on Maternal and Child Nutrition list to develop our intervention and considered the feasibility of scale-up in low resource settings and targeting the first 1000 days of life. Later on, we identified articles that examined the effect of food-assisted maternal and child health and nutrition programs on stunting among children aged 1-24 months in Guatemala. We also significantly reduced the prevalence of stunting at 24 months by 11.1 percentage points. Evidence from Haiti has also shown that food rations and WSB+ given to PLW and children under two can reduce stunting more effectively than providing food rations and WSB+ once the child has become underweight. A protective effect from stunting was also seen in Burundi through food rations and fortified-blended foods in PLW and children under two years of age.

**Added value of this study**

This trial assessed the effectiveness of different nutrition-based supplements during the first 1000 days of life on nutritional outcomes. The intervention package consisted of a preventative nutrition-based approach to reduce stunting in children two years of age in two rural districts in Pakistan. The program focused on the 1000 days window of opportunity, where supplementary nutrition feeding (WSB+ for PLW; LNS for children 6-23 months and critical nutrition, health, and hygiene messages were provided to PLW their children aged 6-23 months. In addition, the program was integrated into the primary healthcare system through the LHW Programme, where LHWs provided the interventions to the targeted PLW and children aged 6-23 months. Our trial findings will generate evidence for guiding future policy and program design at a provincial and national level.

**Implications of all the available evidence**

The findings of the trial revealed that the provision of supplementation during pregnancy and the lactation period is effective in improving linear growth (length and length-for-age z-score), weight, weight-for-age z-score, and underweight (WAZ < -2 SD) at birth and six months of age. Moreover, the provision of WSB+ to PLW and LNS to children 6-23 months effectively reduces the prevalence of stunting and underweight in children at 24 months of age. Children in the intervention group had significantly lower LAZ, WAZ, and WLZ at 24 months compared to those in the control group. This trial and previous research showed that stunting prevention programs are an effective short-term strategy to reduce malnutrition in children due to food insecurity and lack of timely access to adequate age-appropriate nutritious foods in vulnerable countries such as Pakistan.

Alt-text: Unlabelled box

## Introduction

In children, malnutrition (stunting, wasting, and underweight) is prevalent in low- and middle-income countries and is one of the most significant risk factors for deaths in children under five years of age.^1^ Malnutrition also impacts childhood morbidity, leading to delayed cognitive development and physical growth, and is the source of many illnesses later on in their lives.^2^ The nutritional status of children in Pakistan is appalling, with 40.2% of the children under five stunted, 17.7% wasted, and 28.9% underweight, with a higher prevalence in rural areas than in urban areas, particularly in the Sindh province (45.5%).^3^

The first 1000 days of a child's life are the most vulnerable and critical for building the foundations of optimum growth and development of the child.^4^ Nutritional supplementation during the first 1000 days of life have shown improvements in birth and growth outcomes and newborn stunting.^5^^,^^6^ Lack of adequate maternal nutrition during preconception, pregnancy, and postpartum is associated with adverse maternal and child health outcomes, including poor birth outcomes.^4^^,^^7^

Common food-based interventions, including providing supplementary nutrition feeding during pregnancy and the first six months of lactation, followed by complementary feeding for the infant up to two years of age, have proven to be more effective and holistic in targeting nutrient deficiencies in infants.^8^ Balanced protein-energy supplementation, also known as nutrition-based supplements, when given during pregnancy, they have shown to reduce the prevalence of low-birth-weight infants by 32% and a 34% reduction in small-for-gestational-age babies.^9^ The provision of such supplements to pregnant women has also reduced neonatal mortality risk, and increased birth length and weight.^10^^,^^11^ A recent review concluded that Lipid-based nutrient supplements (LNS) plus complimentary feeding compared to no intervention is effective in improving growth outcomes and reducing stunting, moderate underweight, moderate wasting, and anemia among children aged 6-23 months in low‐ and middle‐income countries in Asia and Africa, and more effective if provided over a longer duration of time (over 12 months).^12^

LNS are energy-dense products containing micronutrients and essential fatty acids. They indicated high acceptance and satisfaction with LNS and perceptions that it positively affects child health and development.^13^, ^14^, ^15^, ^16^ When provided as a complementary food for infants, LNS has improved nutrition-related outcomes in children.^17^ The lines-DYAD study found that children randomized to take LNS had greater length, length-for-age z-scores (LAZ), weight, weight-for-age z-scores (WAZ), and reduced incidence of stunting at 18 months compared to the other groups.^18^

Poor knowledge of infant and young child feeding (IYCF) is a significant determinant of stunting among children 6-23 months. This period is considered a critical window of opportunity to introduce an appropriate complementary food to ensure adequate child growth and development.^8^^,^^19^ Improvement in complementary feeding practices can be achieved by increasing the knowledge of mothers and caregivers about age-appropriate diets, and feeding practices through community-based nutrition education interventions.^20^^,^^21^ Poor knowledge about appropriate IYCF practices can lead to inadequate nutritional intakes and adverse impact on child health and development.^21^

The Stunting Prevention Programme was launched in 29 union councils (UCs) of districts Thatta and Sujawal, Sindh Pakistan from 2014 – 2018. The program consisted of the provision of preventative nutrition-based supplements (wheat soya blend plus (WSB+) to pregnant and lactating women (PLW), lipid-based nutrient supplement - medium quantity (LNS-MQ) to children 6–23 months, and behavior change communication messages targeting mothers. In comparison, the control group received routine public health services through primary health facilities. The program was integrated with the primary healthcare system through the Lady Health Worker (LHW) Program, Government of Pakistan. The intervention delivery was administered by LHWs, who play an integral role in Pakistan's maternal and child health services. Each LHW caters to approximately 100 households, and 15–20 LHWs are affiliated with a public-sector health facility in each union council. The provision of specialized nutrition supplementation could not only improve maternal and neonatal health outcomes but also pave the way to prevent stunting, wasting and underweight in children at two years of age. Therefore, we aimed to assess the effectiveness of nutritional supplementation during the first 1000-days of life to reduce the prevalence of stunting in children at 24 months of age.

## Methods

### Study design and participants

We conducted a community-based cluster randomized controlled trial to evaluate the effectiveness of different nutrition-based supplements on nutritional outcomes in both women and their children during the first 1000-days of life. The study was conducted between August 30, 2015, and October 25, 2018, in districts Thatta and Sujawal in Sindh province, Pakistan.

These districts are divided into 9 Talukas and 55 Union Councils (UCs) and have approximately 1.8 million people. Each UC tends to have at least one public healthcare facility. Ethical approval for the study was granted by the Ethics Review Committee of Aga Khan University and the National Bioethics Committee of Pakistan for research including human subjects. All participants included in the study provided written informed consent before enrolment. This study was registered on ClinicalTrials.gov under the registration number NCT02422953.

Pregnant women identified at any stage of their pregnancy were eligible for enrollment in the study. These pregnant women were identified through the LHW family register and meetings with mothers and health care providers in the study area. These enrolled pregnant women were followed up every month throughout the study during their pregnancy and the first six months of lactation. In addition, their live-born infants were followed up till two years of age.

### Randomization and masking

Out of 29 stunting prevention program UCs, 12 UCs were randomly selected and equally assigned to the intervention or control groups through a computer-generated randomization sequence by the data management unit at the Aga Khan University. The unit of randomization was UC. The UCs were matched on coverage of LHWs, the proportion of stunting among children under five years, the number of pregnant women, and population size. The intervention package included a distinctly visible component; therefore, the study participants and data collection team were not masked in the intervention assignment. However, data collection teams different in both groups, and data analysts remained blinded to study arms until the final analysis was completed.

### Procedures

LHWs provided WSB+ to women during their pregnancy and during the first six months of lactation in the intervention group. The WSB+ consisted of partially cooked wheat and soya beans fortified with vitamins and minerals. PLWreceived 5 kg (165g per day) of WSB+ every month for the duration of their pregnancy and through the first six months of lactation. Infants of these mothers were provided LNS from 6 to 23 months of age. LNS was prepared with roasted chickpeas, vegetable oils, dry skimmed milk, sugar, vitamins, and minerals, recommended emulsifier, and antioxidants. The supplementation procurement, storage, and transportation to the health facility were facilitated and managed by, the World Food Programme. The LHWs conducted counseling sessions during supplements distribution, community sessions, and home visits every month. Individual and community essions included messages on maternal nutrition during pregnancy, initiation of breastfeeding within one-hour, exclusive breastfeeding for six months, child nutrition through age-appropriate complementary feeding during 6–24 months, and messages on usage and benefits of supplements. The control group received routine public health services through primary health facilities,including counseling on maternal and child nutrition, health and hygiene. The details of the intervention package, nutritional values of supplements, and other procedures are reported elsewhere.^22^^,^^23^

A total of six data collection teams were hired locally from the study area, and each team was comprised of four female data collectors and a team leader. Data collectors were required to have a minimum high school education, and the team leader required a minimum graduate-level education (14^th^ grade). All data collectors received 6-day training on data collection techniques, anthropometric measurements, and ethical issues with one-day field pilot testing before data collection. Questionnaires were field-tested before the study commenced, and changes and suggestions were incorporated. Each team leader was given a study manual with instructions, methodology, and sampling strategy. The team leader was responsible for coordinating with community leaders and stakeholders, supervising of daily field activities and questionnaire reviews. The teams collected data manually on hard copies of the study questionnaires. In addition, the socio-demographic information, gestational age, reproductive history, antenatal care, morbidities, health-seeking behavior, past exposure to the interventions, and anthropometric data were collected.

Participants were followed up every month to assess their compliance to the intervention, pregnancy outcomes, maternal and infant morbidity, and mortality. Participant recall and the observation of used and unused WSB+ sachets were used to assess compliance during each visit. In addition, Seca Anthropometry Kits were used to measure the anthropometric data of PLW and their infants monthly.

### Outcomes

The primary outcome of this trial was a reduction in the prevalence of child stunting at 24 months of age. Secondary outcomes included mean length, weight, length-for-age Z scores, weight-for-age Z scores, weight-for-length Z scores; the proportion of underweight children (i.e., weight-for-age Z score less than -2), and wasted (i.e., weight-for-length Z scores -2); and improvement in minimum dietary diversity, minimum meal frequency and minimum acceptable diet in children at 24 months of age.

### Statistical analysis

The sample size was calculated to detect a 20% difference in the prevalence of stunting (from 56.2% to 44.9%) among children at 24 months of age in the intervention compared to the control group. We estimated the study power 90%, assumed ∼166 children per cluster, and defined significance at a p-value of 0.05. A total of 1992 children (996 per group) was required from 12 clusters. This sample size included 30% attrition.

Data were entered twice by data entry operators in a Visual FoxPro database. First, anthropometric indices (weight‐for‐age, length‐for‐age, and weight-for‐length z scores) were calculated using WHO growth standards. Stunting, wasting, and underweight was defined as length-for-age z score (LAZ) of <-2 SD, weight-for-length z score (WLZ) of <-2 SD, and weight-for-age z score (WAZ) of <-2 SD, respectively. For impact analyses, we adopted the intention-to-treat approach. The primary outcome was a reduction in the prevalence of stunting among children at 24 months, while secondary outcomes included the difference in length, weight, LAZ, and WAZ at birth and the prevalence of underweight and wasting and improvement IYCF practices at 24 months of age. The growth measurements were reported at birth (including births captured within 28 days), 6, 12, 18, and 24 months.

We fitted generalized linear models (GLM) to estimate the effect of the intervention on both the continuous and the binary outcomes with standard robust variance estimation to consider the clustering effect by the union council. Gaussian and logit link functions were used for continuous and binary outcomes, respectively. The use of GLM for linear and binary outcomes is well established and allows for a straightforward interpretation of average intervention effects with mean differences expressed as a difference in mean scores and percentage points for continuous and categorical outcomes.

For multivariate analysis, the logistic mixed-effect model was used to explore the intervention's impact on the child's stunting status. Clusters and individuals were incorporated as a random effect to model individual heterogeneity within intervention groups. The intervention and time interaction were tested to measure the impact of change in outcome by intervention. Furthermore, the analysis adjusted for maternal age, BMI, gestational age at birth, household hunger, improved drinking water, and toilet facility. Group means with 95% confidence intervals (CIs) were reported. The analysis was carried out in STATA version 17.

### Role of the funding source

The funding agency of this trial had no role in the study design, data collection, data analysis, data interpretation, or writing of the manuscript. The corresponding author had full access to all the data set and full responsibility for submitting it for publication.

## Results

**Figure 1:**
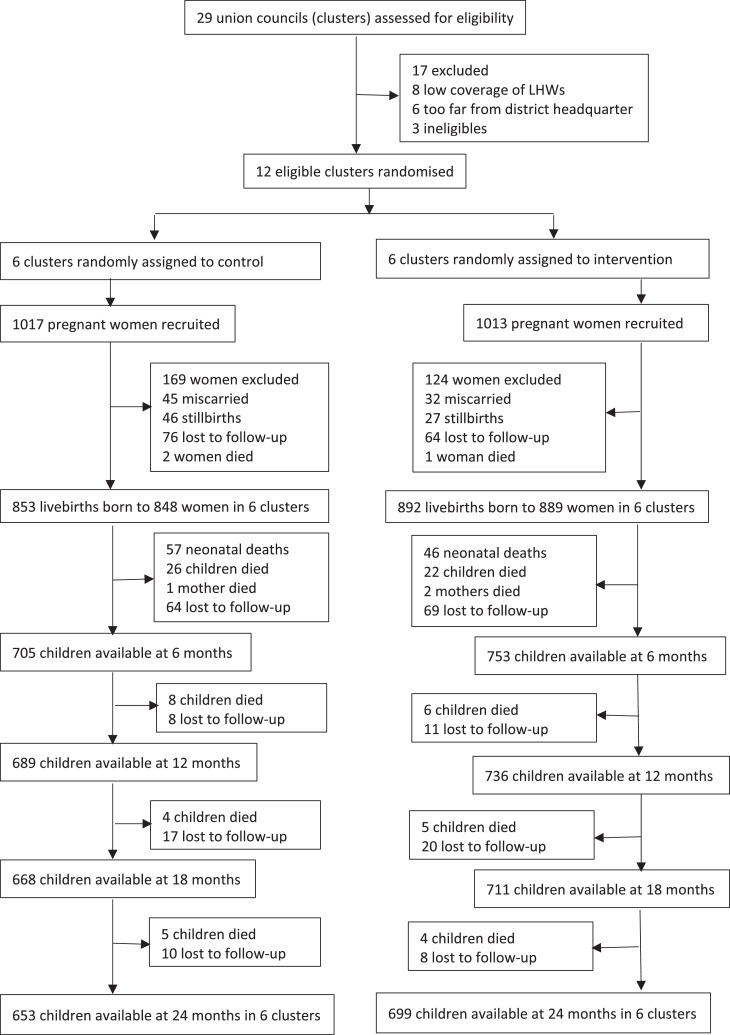
Study profile of the cluster randomized controlled trial of monthly supply of WSB+ during pregnancy and lactation and LNS-MQ for children 6-23 months in Pakistan. LNS-MQ, lipid-based nutrient supplement - medium-quantity; WSB+, wheat soya blend plus.

Two thousand thirty pregnant women were enrolled in the study at a mean gestational age of 21 weeks between August 30, 2014, and May 25, 2016. Monthly follow-ups were conducted between October 1, 2014, and October 25, 2018. All clusters were retained in the study. Between recruitment and pregnancy outcome, 293 (14%) women were excluded from the study, 77 (3.8%) miscarried, 73 (3.6%) stillbirths, 140 (6. 9%) lost to follow-up, and 3 (0.1%) women died. Out of the 1745 live births, 17 women gave birth to twins. A total of 103 (5.9%) neonatal deaths were reported, and 48 (2.7%) children died between 1 to 6 months of age and 32 (1.8%) children between 6 to 24 months of age. In total, 1352 children (653 in the control group; 699 in the intervention group) were available at 24 months of age for final analysis (Figure 1).

There were no significant differences between the intervention and control groups in maternal and household characteristics at baseline (Table 1). The mean compliance of WSB+ during pregnancy was 60% (55.2 - 64.5), compliance during the first six months of lactation was 59% (55.8 - 63.2), and mean compliance of LNS among children 6-24 months was 52% (46.2 - 57.2) (Table 2).

**Table 1:** Maternal and household baseline characteristics.^a^

Characteristics	Control	Intervention	*P*-values
	(*n* = 1017)	(*n* = 1013)	
**Maternal characteristics**			
Age (years)^b^	29.7 (28.4 to 31.0)	29.2 (28.6 to 29.7)	0.416
Gestational age (weeks)^b^	21.4 (19.1 to 23.7)	21.5 (20.2 to 22.8)	0.920
Height (cm)^b^	154.4 (153.9 to 154.9)	154.8 (154.4 to 155.1)	0.183
Weight (kg)^b^	49.2 (47.9 to 50.5)	50.4 (49.1 to 51.6)	0.166
MUAC (cm)^b^	24.1 (23.7 to 24.4)	24.4 (24.1 to 24.6)	0.175
Mean BMI (kg/m^2^)^b^	20.6 (20.1 to 21.2)	21.0 (20.5 to 21.5)	0.266
Underweight (BMI <18.5)^c^	23.9 (19.4 to 29.0)	20.0 (16.2 to 24.5)	0.241
Normal (BMI 18.5-24.9)^c^	66.9 (64.7 to 69.0)	70.8 (65.7 to 75.4)	0.174
Overweight/obese (BMI ≥ 25)^c^	9.2 (6.1 to 13.8)	9.2 (5.9 to 14.0)	0.981
**Household characteristics**^c^			
Improved water	88.5 (80.8 to 93.4)	87.7 (80.5 to 92.5)	0.833
Improved toilet facility	26.0 (13.1 to 44.9)	45.1 (28.9 to 62.4)	0.104
Availability of soap and water	37.5 (31.8 to 43.7)	39.0 (31.5 to 47.0)	0.749
**Household hunger scale**^c^			
None or light hunger (0-1 score)	89.0 (83.3 to 92.9]	90.9 (86.8 to 93.8]	0.482
Moderate hunger (2-3 scores)	10.4 (7.0 to 15.2]	8.9 (6.1 to 12.8]	0.530
Severe hunger (4-6 scores)	0.6 (0.2 to 1.9]	0.2 (0.1 to 0.7]	0.259

a The analysis is accounted for clustering.

b Data present as mean and 95% CIs.

c Data present as percentage and 95% CIs. Abbreviations: BMI, body mass index; CI, confidence interval; CM, centimeters; Kg, kilogram; MUAC, mid upper arm circumference.

**Table 2:** Compliance of WSB+ during pregnancy and first six months of lactation period and LNS in children during 6-24 months of age.^a^

Indicators	Pregnancy	Lactation	Children 6-24 m
	(*n =*1013)	(*n =*830)	(*n =*701)
**Mean days observed**	120.7 (112.1 to 129.4)	153.9 (144.9 to 162.9)	445.1 (435.9 to 454.4)
**Mean days supplement received**	95.2 (87.7 to 102.7)	84.5 (74.3 to 94.7)	233.2 (207.7 to 258.6)
**Mean compliance (%) (days consumed/days observed**^a^**100)**	59.9 (55.2 to 64.5)	59.5 (55.8 to 63.2)	51.7 (46.2 to 57.2)

a Data present as percentage and 95% CIs.

There was a significant difference in mean length (49.4 cm vs 48.9 cm, *p =* 0.027), weight (3.1 kg vs 3.0 kg, *p =* 0.013), length for age z-scores (-1.2 vs -1.5, *p =* 0.004) and weight for age z-scores (-1.2 vs -1.5, *p =* 0.015) among infants in the intervention group as compared to control group. Similarly, a significant difference was noted in the prevalence of underweight among infants in the intervention group as compared to control group (24.2% vs 31.2%, *p =* 0.013). No significant difference was noted in the prevalence of stunting (27.9% vs 31.7%, *p =* 0.195), wasting (12. 3% vs 14.9%, *p =* 0. 316), and weight for length z-scores (-0.7 vs -0.7, *p =* 0.647) among infants in both groups (Table 3).

**Table 3:** Nutritional status of infants within 28 days of life.^a^

	Control	Intervention	Mean Difference	*p*-value
	(*n =*561)	(*n =*559)	% (95% CI)	
Length (cm)^b^	48.9 (48.5 to 49.3)	49.4 (49.0 to 49.7)	0.50 (0.07 to 0.92)	0.027
Weight (kg)^b^	3.0 (2.9 to 3.0)	3.1 (3.0 to 3.2)	0.11 (0.03 to 0.20)	0.013
Length-for-age Z score^b^	-1.5 (-1.7 to -1.4)	-1.2 (-1.4 to -1.1)	0.29 (0.11 to 0.46)	0.004
Weight-for-age Z score^b^	-1.5 (-1.6 to -1.4)	-1.2 (-1.5 to -1.0)	0.26 (0.06 to 0.46)	0.015
Weight-for-length Z score^b^	-0.7 (-0.8 to -0.6)	-0.7 (-1.0 to -0.4)	0.05 (-0.19 to 0.30)	0.647
Stunting (LAZ <-2)^c^	31.7% (25.9 to 38.2)	27.9% (23.9 to 32.3)	-3.82% (-9.92 to 2.28)	0.195
Under-weight (WAZ <-2)^c^	31.2% (26.6 to 36.2)	24.2% (20.3 to 28.6)	-6.95% (-12.14 to -1.76)	0.013
Wasted (WLZ <-2)^c^	14.9% (11.5 to 19.1)	12.3% (7.7 to 19.0)	-2.61% (-8.08 to 2.86)	0.316

a Analysis is based on measurements for infants within 28 days of birth and accounted for clustering.

b Mean and 95% CIs as reported.

c Data present as percentage and 95% CIs. Abbreviations: CM, centimeters; Kg, kilogram; LAZ, length-for-age z score; WAZ, weight-for-age z score; WLZ, weight-for-length z score.

### Primary outcome

The primary outcome of this trial was a reduction in the prevalence of stunting in children at 24 months of age. After adjustment for clustering, a significant difference in the prevalence of stunting was noted among children at 6 months (absolute difference, -12.5%, *p =* 0.002), 12 months (absolute difference, -16.5%, *p =* 0.001), 18 months (absolute difference, -13.4%, *p =* 0.008) and at 24 months of age (absolute difference, -10.2%, *p =* 0.0.017) in the intervention group compared with control group (Table 4). Multiple covariate adjustments did not change the stunting status among children at baseline, 6, and 18 months but the difference diminished at 24 months of age (Table 7).

**Table 4:** Prevalence of stunting, underweight, and wasting among children at 6, 12, 18, and 24 months of age.^a^

	Control	Intervention	Mean Difference	*P* - value
**Stunting (LAZ <-2)**^c^				
6 months	33.7 (29.2 to 38.2)	21.2 (16.9 to 25.6)	-12.5 (-19.1 to -5.8)	0.002
12 months	50.8 (45.4 to 56.3)	34.4 (30.1 to 38.6)	-16.5 (-23.8 to -9.1)	0.001
18 months	62.2 (55.5 to 68.9)	48.8 (43.7 to 53.9)	-13.4 (-22.3 to -4.4)	0.008
24 months	59.9 (54.9 to 64.9)	49.7 (44.1 to 55.3)	-10.2 (-18.2 to -2.3)	0.017
**Underweight (WAZ <-2)**^c^				
6 months	49.0 (44.3 to 53.7)	37.8 (33.6 to 42.0)	-11.2 (-17.9 to -4.5)	0.004
12 months	57.7 (52.0 to 63.4)	42.3 (36.3 to 48.3)	-15.4 (-24.2 to -6.6)	0.003
18 months	56.2 (51.4 to 60.9)	41.3 (35.9 to 46.7)	-14.8 (-22.5 to -7.2)	0.002
24 months	58.0 (53.8 to 62.3)	44.4 (39.9 to 48.9)	-13.7 (-20.3 to -7.0)	0.001
**Wasting (WHZ <-2)**^c^				
6 months	29.5 (24.7 to 34.4)	24.3 (19.8 to 28.7)	-5.3 (-12.3 to 1.7)	0.123
12 months	36.2 (33.0 to 39.4)	24.4 (17.6 to 31.1)	-11.9 (-19.8 to -3.9)	0.008
18 months	32.6 (29.6 to 35.6)	21.9 (15.7 to 28.2)	-10.7 (-18.0 to -3.3)	0.009
24 months	27.5 (23.7 to 31.4)	20.6 (15.0 to 26.2)	-6.9 (-14.1 to 0.3)	0.057

a The analysis is accounted for clustering using a linear regression model.

c Data present as percentage and 95% CIs. Abbreviations: CI, confidence interval.

### Secondary outcomes

A significant difference in the prevalence of underweight was noted among children at 6 months (absolute difference, -11.2%, *p =* 0.004), 12 months (absolute difference, -15.4%, *p =* 0.003), 18 months (absolute difference, -14.8%, *p =* 0.002) and at 24 months of age (absolute difference, -13.7%, *p =* 0.001). Furthermore, a significant difference in the prevalence of wasting was also noted among children at the age of 12 months (absolute difference, -11.9%, *p =* 0.008), and 18 months (absolute difference, -10.7%, *p =* 0.009), but no difference was noted at the age of 6 months (absolute difference, -5.3%, *p =* 0.123), and at 24 months of age (absolute difference, -6.9%, *p =* 0.057) in both groups (Table 4).

We also detected effect of intervention package on length-for-age z-scores at 6 months (mean difference (MD) 0.4; 95% CI 0.3 to 0.6), *p =* <0.001); 12 months (MD 0.3; 95% CI 0.1 to 0.5), *p =* 0.003); 18 months (MD 0.4; 95% CI 0.2 to 0.6), *p =* 0.003); and at 24 months of age (MD 0.3; 95% CI 0.1 to 0.6), *p =* 0.008) among children in the intervention group compared with control group. Similarly, differences in weight-for-age z-scores were significant at 6 (*p =* <0.001), 12 (*p =* 0.001), 18 (*p =* 0.003) and 24 months of age (*p =* 0.011). Differences in mean z-scores for weight-for-length was also significant at 12 (*p =* 0.004), 18 (*p =* 0.014) and 24 months of age (*p =* 0.033) in the intervention group compared with control group (Table 5).

**Table 5:** LAZ, WAZ, and WLZ at 6, 12, 18, and 24 months of age.^a^

	Control	Intervention	Mean Difference	*P* - value
	Mean (95% CI)	Mean (95% CI)	(95% CI)	
**Length-for-age Z score**^b^				
6 months	-1.6 (-1.7 to -1.5)	-1.1 (-1.2 to -1.0)	0.4 (0.3 to 0.6)	<0.001
12 months	-2.1 (-2.2 to -1.9)	-1.7 (-1.8 to -1.6)	0.3 (0.1 to 0.5)	0.003
18 months	-2.4 (-2.6 to -2.3)	-2.1 (-2.2 to -1.9)	0.4 (0.2 to 0.6)	0.003
24 months	-2.4 (-2.6 to -2.2)	-2.0 (-2.2 to -1.9)	0.3 (0.1 to 0.6)	0.008
**Weight-for-age Z score**^b^				
6 months	-2.0 (-2.1 to -2.0)	-1.6 (-1.8 to -1.5)	0.4 (0.2 to 0.6)	<0.001
12 months	-2.2 (-2.4 to -2.1)	-1.8 (-2.0 to -1.6)	0.4 (0.2 to 0.6)	0.001
18 months	-2.3 (-2.4 to -2.2)	-1.9 (-2.1 to -1.7)	0.4 (0.2 to 0.6)	0.003
24 months	-2.3 (-2.4 to -2.2)	-1.9 (-2.1 to -1.7)	0.3 (0.1 to 0.6)	0.011
**Weight-for-length Z score**^b^				
6 months	-1.4 (-1.5 to -1.3)	-1.2 (-1.4 to -1.0)	0.2 (-0.0 to 0.4)	0.075
12 months	-1.6 (-1.7 to -1.6)	-1.3 (-1.5 to -1.1)	0.3 (0.1 to 0.5)	0.004
18 months	-1.6 (-1.6 to -1.5)	-1.3 (-1.5 to -1.1)	0.3 (0.1 to 0.5)	0.014
24 months	-1.4 (-1.5 to -1.4)	-1.2 (-1.4 to -1.0)	0.2 (0.0 to 0.5)	0.033

a The analysis is accounted for clustering using a linear regression model.

b Data present as percentage and 95% CIs. Abbreviations: LAZ, length-for-age z score; WAZ, weight-for-age z score; WLZ, weight-for-length z score.

Table 6 shows the effect of the intervention package on minimum dietary diversity, minimum meal frequency, and minimum acceptable diet among children at 6, 12, 18, and 24 months of age. We found a significant improvement in minimum dietary diversity (*p =* 0.018) and minimum acceptable diet (*p =* 0.004) in the intervention versus the control group at 24 months. However, the improvement in minimum meal frequency among children at 24 months did not differ between the intervention and control groups (*p =* 0.297).

**Table 6:** Minimum dietary diversity, meal frequency, and acceptable diet among children at 6, 12, 18, and 24 months of age.^a^

IYCF practices	Control	Intervention	*p*-value
	Mean (95% CI)	Mean (95% CI)	
**Minimum dietary diversity**^b^			
6 months	0.3 (0.1 to 2.1)	1.1 (0.6 to 2.1)	0.106
12 months	2.5 (1.4 to 4.6)	4.9 (3.4 to 7.2)	0.049
18 months	3.3 (1.9 to 5.6)	8.4 (5.5 to 12.6)	0.015
24 months	5.1 (2.7 to 9.3)	11.9 (8.1 to 17.1)	0.018
**Minimum meal frequency**^b^			
6 months	9.0 (6.9 to 11.5)	10.1 (6.4 to 15.7)	0.616
12 months	20.8 (15.4 to 27.5)	26.8 (23.5 to 30.4)	0.085
18 months	42.5 (35.6 to 49.7)	49.0 (42.8 to 55.3)	0.155
24 months	70.1 (62.8 - 76.4)	75.0 (67.1 to 81.5)	0.297
**Minimum acceptable diet**^b^			
6 months	0.3 (0.1 to 2.1)	1.1 (0.6 to 2.1)	0.106
12 months	2.4 (1.2 to 4.6)	4.8 (3.1 to 7.2)	0.062
18 months	2.5 (1.4 to 4.3)	6.6 (4.1 to 10.7)	0.023
24 months	1.6 (0.9 to 2.8)	6.3 (4.1 to 9.6)	0.004

a The analysis is accounted for clustering using a linear regression model.

b Data present as percentage and 95% CIs.

**Table 7:** Estimated stunting status among children at 0, 6, 12, 18 and 24 months of age by intervention group.^a^

Stunting	Unadjusted^a^			Adjusted		
	Control	Intervention	*P*-value^c^	Control	Intervention	*P*-value^d^
	% (95% CI)	% (95% CI)				
0 month^b^	15.5 (10.6 to 20.5)	12.5 (8.4 to 16.6)	Ref.	16.3 (11.4 to 21.2)	14.6 (10 to 19.2)	Ref.
6 months	15.9 (11.2 to 20.6)	7.6 (5 to 10.2)	0.035	16.6 (11.9 to 21.3)	9.1 (6.1 to 12.2)	0.035
12 months	45.4 (36.9 to 53.9)	23.2 (17 to 29.5)	0.006	44.3 (36.6 to 52.1)	25.9 (19.5 to 32.3)	0.006
18 months	71.1 (64 to 78.3)	46.1 (37.8 to 54.3)	0.004	68.4 (61.4 to 75.3)	47.9 (40.3 to 55.5)	0.004
24 months	66.2 (58.6 to 73.8)	49.4 (41.3 to 57.5)	0.101	63.7 (56.4 to 70.9)	50.9 (43.5 to 58.3)	0.105

a The analysis is accounted for clustering using a linear regression model.

b Based on measurements for children enrolled within 28 days of birth.

c P-value for interaction of intervention with time.

d Adjusted for maternal age, body mass index, maternal gestational age, improved drinking water, improved toilet facility and household hunger scale. ^€^ Data present as percentage and 95% CIs.

## Discussion

To the best of our knowledge, this is the first large, community-based cluster randomized controlled trial to test the effectiveness of nutritional supplementation and nutrition education during the first 1000-days of life to reduce child stunting in Pakistan. We evaluated the effectiveness of WSB+ provided during pregnancy and the first six months postpartum and LNS-MQ for their infants from 6 to 24 months of age. We found a significant 17% reduction in the prevalence of stunting among children at 24 months of age in the intervention compared to the control group. We also found a significant reduction (23%) in the prevalence of underweight but not for wasting among children at 24 months of age. In addition, we found that newborns in the intervention group had greater length, weight, LAZ, WAZ, and underweight than the control group. By 24 months, the mean differences were evident for LAZ (+0.3), WAZ (+0.3), and WLZ (+0.2) in the intervention group compared with the control group. In addition, a significant improvement in minimum dietary diversity and minimum acceptable diet was noted among children at 24 months of age in the intervention compared with the control group.

We can compare our results with four trials, which provided supplementation to women during pregnancy, six months postpartum, and their infants starting from 6 months of age.^18^^,^^24^, ^25^, ^26^ In rural Niger, prenatal supplementation with multiple micronutrient supplements or medium-quantity lipid-based nutrient supplements reported no effect on child LAZ, WAZ or WLZ at 24 months of age.^24^ In the Malawi trial, the provision of small-quantity lipid-based nutrient supplements (SQ-LNS) to mothers in pregnancy and six months postpartum and to their infants from 6-18 months of age had no effect on mean length, weight, MUAC and prevalence of stunting at 18 months of age.^25^ Several explanations are possible for these findings; a significant reason for the difference in results between the Malawi study and our study may be the study context. In Malawi, child linear growth may have been restricted due to the high prevalence of asymptomatic infections, environmental enteropathy, and the short stature of mothers; between 11% and 15% of women were HIV positive at baseline, compared to no data on HIV in our study. In Ghana, SQ-LNS provided during pregnancy, lactation, and to infants from 6-18 months of age significantly improved linear growth (+0.28 LAZ) and reduced stunting (6.2 percentage points).^18^ Similarly, in Bangladesh, small-quantity LNS provided to women during pregnancy; first six months postpartum and LNS to their offspring from 6 to 24 months improved mean LAZ at 24 months (+0.13) between the children exposed to both prenatal and postnatal LNSs and reduction in stunting prevalence at 18 months (21%) in the LNS-LNS group compared with the IFA-MNP group.^26^ Our trial results are almost similar to those from Bangladesh and Ghana on mean length, LAZ, weight, and weight-for-age z score and stunting.^18^^,^^26^ At 24 months, the significant difference for LAZ was (+0.3), WAZ (+0.3), and WLZ (+0.2) in the intervention group compared with the control group. Similarly, the reduction in the prevalence of stunting was (13.4 percentage points), and underweight was (14.8 percentage points) at 18 months of age.

Our study findings are consistent with the impact of complementary feeding research studies,^5^^,^^27^^,^^28^ For example, our intervention increased linear growth and proportion of children stunted at 24 months of age was significantly lower in the intervention group (49.7%) than in the control group (59.9%), or 10.2 percent points. In addition, although we could not separate the effects of nutrition education from nutrient-based supplements, other studies showed that both components are essential intervention combinations.^29^^,^^30^ Similarly, a study in Burkina Faso reported a 0.5-cm increase in birth length in the intervention group who received prenatal LNSs (373 kcal/d) compared with those who received MMNs.^31^ We also found a 0.50 cm increase in length among newborns in the intervention group compared with the control group.

Children exposed to intervention during the first 1000-days experienced an increase of 0.3 SD in length-for-age Z scores at 24 months of age compared to the control group. The effect on the length-for-age Z score seen in this study is in line with those reported in trials of nutrition education without supplementary feeding and higher than those reported in previous trials of nutrition education and hygiene promotion globally and in India.^32^ A 2013 systematic review of five trials found that complementary feeding education led to a 0·23 increase in height-for-age Z score in children younger than two years.^33^

Our nutrition education intervention improved self-reported minimum dietary diversity and minimum acceptable diet at 6, 12, 18, and 24 months of age. However, the proportion of minimum dietary diversity and minimum acceptable diet remained just below the recommended diet for young children by the World Health Organization.^34^ Results from four randomized controlled trials in Africa, where a small quantity LNS (SQ-LNS) was provided to women during pregnancy and six months postpartum and to their infants from 6 to 18 months of age, concluded that the provision of SQ‐LNS did not negatively impact self‐reported IYCF practices and may have positively impacted frequency of feeding.^18^ Other studies from Kenya, Uganda, India, and Malawi with community-based nutrition education messages and home-visit counseling also reported improvements in dietary diversity.^35^, ^36^, ^37^, ^38^, ^39^, ^40^

Our study had many strengths, such as the intervention package delivered through the existing government-supported LHW program, cluster-randomized controlled study design with the active control group, and consistent monthly follow-ups to trace pregnancy outcomes, IYCF practices, and children's growth at different timepoints. Furthermore, all anthropometrists were well trained and standardized, and we undertook all efforts to ensure data quality. However, there were some inevitable limitations in the study design. First, the study could not be blinded, leading to bias. However, data collection was standardized, interviews were structured, and data collectors rotated between intervention and control groups to limit any bias that might result from the same team always interviewing intervention or control participants.

Nevertheless, knowledge of the group could have influenced data collectors' interpretation of responses or the recording of dietary-recall data. Still, this knowledge is unlikely to have affected weight or length measurements. Second, dietary diversity and adherence to supplement intakes were collected via maternal reporting rather than direct observation, leading to the over-reporting of desirable practices. However, the differences in dietary patterns between the intervention and control group were not consistent, which suggests that bias is unlikely. Third, sharing supplements with other family members may have a limited impact on children's linear growth and nutritional status at 24 months.

In conclusion, the provision of WSB+ and LNS-MQ and nutrition education during the first 1000 days of life may be effective for improving the linear growth of infants and reducing the prevalence of stunting among children at two years of age in similar settings. However, in populations with a higher prevalence and more complex etiology of stunting, the impact of WSB+ and LNS-MQ should be tested more broadly in local programs that integrate nutrition into routine interventions to reduce childhood stunting.

## Contributors

SBS conceptualized the study. ZAB provided technical inputs. SBS, GNK, and SA coauthored the original protocols and contributed to the design and implementation of the trial and data interpretation. GNK led the data collection teams' training and the study's implementation. AR and MS are involved in data management and data analysis. All authors contributed to, reviewed, and approved this final manuscript.

## Data sharing statement

A protocol has been published. Anonymized participant data and a data dictionary are available to be shared subject to a data sharing agreement. Data will be shared following a request and approval by the corresponding author.

## Declaration of interests

We declare no competing interests.
